# Preventing atrial fibrillation with SGLT2 inhibitors: time, sex, and substrate

**DOI:** 10.1093/ehjcvp/pvaf066

**Published:** 2025-09-08

**Authors:** Paschalis Karakasis, Antonios P Antoniadis, Nikolaos Fragakis

**Affiliations:** Second Department of Cardiology, Aristotle University of Thessaloniki, General Hospital Hippokration, 54642 Thessaloniki, Greece; Second Department of Cardiology, Aristotle University of Thessaloniki, General Hospital Hippokration, 54642 Thessaloniki, Greece; Second Department of Cardiology, Aristotle University of Thessaloniki, General Hospital Hippokration, 54642 Thessaloniki, Greece

We read with great interest the recent meta-analysis by Fedele *et al*.^1^ evaluating the role of sodium–glucose co-transporter 2 (SGLT2) inhibitors in the prevention of atrial fibrillation (AF) across a broad spectrum of cardiovascular disorders. The authors are to be commended for conducting a timely and methodologically sound synthesis that reinforces the AF-preventive potential of SGLT2 inhibitors, particularly in patients without heart failure or with heart failure with reduced ejection fraction.

We particularly appreciate the authors’ emphasis on the diminished benefit observed in patients with HFpEF or HFmrEF—an observation that aligns with emerging mechanistic and imaging data suggesting more advanced atrial remodeling and fibrosis in these phenotypes. This likely reflects a narrower therapeutic window for upstream modulation in the presence of established atrial cardiomyopathy, as indicated by the attenuated effects observed in HFpEF/HFmrEF on the meta-regression of the present meta-analysis,^1^ and by studies demonstrating early, reversible improvements in left atrial mechanics and filling with SGLT2 inhibition—benefits that appear less pronounced once structural remodeling is advanced.^2-4^ These findings prompt a critical question: Should left atrial structural and functional parameters, such as strain or fibrosis burden, serve as more sensitive markers of treatment response than left ventricular ejection fraction alone^5^? Future patient-level analyses and imaging-based studies may help refine this paradigm.

Several additional considerations may enhance the translational value of the present findings. First, the lack of sex-stratified pooled data is noteworthy. Given well-documented sex-based differences in AF pathophysiology, atrial substrate characteristics, and responsiveness to pharmacologic interventions^6-9^—particularly among patients with HFpEF—sex-disaggregated outcomes could reveal clinically meaningful heterogeneity. Since trial-level sex distribution data are likely available, the authors are encouraged to perform a meta-regression based on female enrollment proportion to assess whether sex modifies the effect of SGLT2 inhibitors on AF prevention.

Second, the wide variability in trial follow-up durations—ranging from a few weeks to several years—introduces uncertainty about the timing and durability of benefit. Whether the observed reduction in AF reflects early hemodynamic improvements^4^ or more delayed structural remodeling remains unclear. A temporal stratification of outcomes would have helped elucidate this dynamic.

Third, many trials did not clearly distinguish incident from recurrent AF. Inconsistent characterization of baseline rhythm status and arrhythmic burden therefore obscures whether the observed benefit reflects primary prevention or secondary suppression. Among studies enrolling patients with prior AF, both the operational definition of recurrence and the intensity of rhythm surveillance varied widely, which could dilute or mask secondary-prevention effects. Harmonized, burden-based endpoints and standardized monitoring protocols are needed to resolve this uncertainty.

Lastly, the authors’ observation that the protective effect of SGLT2 inhibitors is attenuated in the presence of hypertension or prior AF underscores the complex and multifactorial nature of arrhythmogenesis. As metabolic therapies are increasingly considered for upstream rhythm control, careful phenotyping to identify ‘responder’ endotypes will be essential to support a precision-medicine approach.

In summary, this meta-analysis offers a valuable contribution to the evolving landscape of cardiometabolic therapy in arrhythmia prevention. Future trials enriched for imaging biomarkers, AF burden quantification, and patient-level risk stratification will be instrumental in guiding the selective use of SGLT2 inhibitors in AF management.

## Data Availability

No data were generated in this study.
